# The Mediating Role of Place Attachment Dimensions in the Relationship Between Local Social Identity and Well-Being

**DOI:** 10.3389/fpsyg.2021.645648

**Published:** 2021-08-02

**Authors:** Fridanna Maricchiolo, Oriana Mosca, Daniele Paolini, Ferdinando Fornara

**Affiliations:** ^1^Department of Education Science, University of Roma Tre, Rome, Italy; ^2^Department of Education, Psychology, Philosophy, University of Cagliari, Cagliari, Italy

**Keywords:** well-being, interdependent happiness, place attachment, social relations, lack of resources, place identity

## Abstract

Well-functioning communities provide a range of material and psychological resources that enhance well-being. The degree to which individuals see themselves as part of the local social group, or local social identity, i.e., the social identification with the community of the place where people are living, may play an important role in enhancing happiness and well-being, as well as relationships of people with their own living environment, i.e., place attachment. We hypothesized that local social identity influences well-being *via* specific components of place attachment to the residential city/town, i.e., place identity, social relations, and lack of resources (which is the opposite of place dependence). We measured local social identity, individual well-being, interdependent happiness, and place attachment in a sample of *N* = 375 participants. We tested our hypotheses by conducting a series of mediation analyses with local social identity as an independent variable, individual well-being and interdependent happiness as dependent variables, and place attachment subfactors, i.e., place identity, social relations, and lack of resources, as mediators. Results showed that the relation between local social identity and both individual well-being and interdependent happiness was positively mediated by place identity and social relations, while the lack of resources emerged as a negative mediator only in the relation between local social identity and individual well-being (not for interdependent happiness). Practical implications and future developments are discussed.

## Introduction

Well-functioning social communities provide a range of material and psychological resources that enhance well-being. Recent research in social psychology has shown that a variety of physical and mental health outcomes are derived from meaningful belonging in social groups within a varied range of contexts, and the local community is one of them (Bowe et al., 2020). Belonging to social groups is a basic psychological need of people because it allows them to preserve security, well-being, and high self-esteem (Baumeister and Leary, 1995). Therefore, people are strongly motivated to belong to social groups, and when their belongingness is threatened, for example, by exposure to ostracism episodes, strong negative consequences follow (see Paolini, 2019).

As postulated by the Social Identity Theory (SIT, Tajfel and Turner, 1979), individuals may thus define themselves either in terms of their belongingness, emphasizing what makes them similar and interchangeable with others, or in terms of their individual characteristics, emphasizing what makes them unique (Tajfel and Turner, 1979). A recent approach, the Social Identity Approach to well-being (Jetten et al., 2017; Haslam et al., 2018), stemming from SIT and Self-Categorization Theory (Turner et al., 1987), suggested that the social dimensions of the self (i.e., the multiple effects—cognitive, emotional, and behavioral—of the sense of “we-ness” derived from group membership) are fundamental in shaping our social world and that the pivotal psychological process connecting social relationships with health and well-being is meaningful social identification, i.e., a subjective sense of belonging (Sani et al., 2012). Social cure research (Haslam et al., 2018) has provided a valuable framework for the study of local community processes. The group with which people identify is a social aggregate most people can claim some access to, and it is as valuable in terms of life satisfaction as other important social identifications (e.g., family; Wakefield et al., 2017). Social identity plays a crucial role at least on two levels of well-being: on the one hand, it is related to the individual well-being level (e.g., satisfaction with life; Diener et al., 1985), and on the other hand, it also could impact the interdependent happiness level, i.e., the happiness based on social relationships, that is, on the relational nature of human beings (Hitokoto and Uchida, 2015; Krys et al., 2019).

We want to underline that the concept of local social identity is different from community attachment, a construct proposed by Hummon (1992) that can be conceptualized as subjective interpretation and the affective reaction of a person to the place in which he/she resides. Hummon (1992) described five ways in which people may relate to their places of residence: two types of rootedness (every day and ideological), which are described in positive terms, and three types of sentiments (alienation, relativity, and placelessness), which are described in negative terms, like estrangement, dislike, and indifference (Lewicka, 2011a,b). People–place relations indeed can have either a positive valence or a negative valence, implying not only a “salutogenic” role but also harmful effects on well-being. On the other hand, well-being, as well as happiness, represents a high value and an important goal of society (Lu and Gilmour, 2004), and it is the result of the accommodations that occur over time and through dynamic interactions of personal, social, and environmental structures and processes (White, 2017; Maricchiolo et al., 2021).

Thus, the social relationships that people establish with closer individuals, social structures, physical environments, as well as with the communities in which people are living (Maricchiolo et al., 2020), represent the “social core” that contributes to maintaining an adequate level of their health and well-being (Haslam et al., 2009; Haslam and Loughnan, 2014; Jetten et al., 2014, 2017).

In order to analyze the connection between individuals, groups, communities, and their living environments, we have focused on the key construct of place attachment, which has been developed in the environmental psychology domain. It concerns those affects, emotions, and feelings that arise from our experience of places (e.g., see Low, 1992; Hidalgo and Hernandez, 2001; Korpela, 2012; Lewicka, 2014; Manzo and Devine-Wright, 2020), where the “place” includes both a physical and a social component (Brown and Perkins, 1992; Hidalgo and Hernandez, 2001; Scannell and Gifford, 2010). Moreover, place attachment also concerns the extent to which the environment satisfies personal needs (Giuliani, 2003), i.e., a functional aspect that has to do with the availability of resources (Scopelliti and Tiberio, 2010). This latter aspect is included in the construct of place dependence, which has been defined as a “functional” connection reflecting the degree to which the physical setting provides conditions to support an intended use (Raymond et al., 2010).

In this study, we followed the conceptualization of place attachment consisting of place identity and place dependence (e.g., Williams and Vaske, 2003) and also social bonds (e.g., Kyle et al., 2005). About place identity, it refers to a substructure of the self that encompasses cognitions, emotions, and behavioral tendencies related to socialization of people with their physical environment (Proshansky et al., 1983).

In most literature on the topic, the analyzed place of attachment is the residential place, with a spatial focus ranging from micro- to macro-levels, i.e., home, the neighborhood, the town/city, or even broader levels. Among such levels, the residential neighborhood has been the prominent place of analysis (Lewicka, 2011b), while less attention has been devoted to the town or city level.

There are also some studies addressing the relationship between place attachment and community participation and well-being. Manzo and Perkins (2006) identified place attachment and participation in neighborhood protection as affective and behavioral place-related community dimensions, respectively. Keyes (1998) showed that social contribution (i.e., the feeling of being a vital member of society, with something of value to contribute) is a specific dimension of social well-being. Similarly, Rollero and De Piccoli (2010) found that attachment to the city is a positive predictor of social well-being and of the social contribution dimension. A positive perception of the living place is a powerful predictor of well-being also for specific populations, such as mentally ill persons (Wright and Kloos, 2007) and the elderly (Fornara et al., 2019), as well as college students, who have to face relocation problems (Scopelliti and Tiberio, 2010). A mediation role of place attachment in the relationship between local civic engagement and personal neighborhood connectedness was found by Buta et al. (2014) with residents living in the area of a national park and also emerged with adolescents (Lenzi et al., 2013). More recently, Larson et al. (2018) found that a stronger place attachment promotes both higher community involvement and higher engagement in place-protective behaviors among hunters, bird-watchers, and property owners. These studies suggest that individuals more attached to the place in which they live are likely to contribute more to the local well-being, through civic activism and the protection of their environment.

The aforementioned literature yields some mixed insights on the connection between place and well-being and shows a relationship between place attachment and satisfaction with life and social well-being. Since the Interdependent Happiness Scale was proposed only in recent years (Hitokoto and Uchida, 2015), to our knowledge, there are no existing studies addressing the relationship between place attachment and happiness based on social relationships. It is important to incorporate a relational-oriented approach to happiness and well-being that complements the individualistic approach to well-being (i.e., based on individualistic-centered measures like the Satisfaction with Life Scale) in people–environment studies. Moreover, empirical evidence on the link between local social identity and different forms of well-being is still substantially lacking. Uncovering the impact on different types of well-being of successful community identities, through place attachment components, is therefore essential to progressing the community development agenda (Bowe et al., 2020).

## The Present Study

Based on these premises, this study aimed to understand whether the relation between social identification of people toward their local community and their level of well-being, in terms of life satisfaction and interdependent happiness, is mediated by place identity, place dependence, and social bonds, i.e., those place attachment components, included in many studies addressing this construct (e.g., Kyle et al., 2005; Raymond et al., 2010; Scopelliti and Tiberio, 2010; Ramkissoon et al., 2013; Chen et al., 2018).

Therefore, as a first step, we verified the three-factor structure of place attachment, and then, in an explorative vein, we tested their mediational role on the relation between local social identification of people and their levels of individual and interdependent well-being. Thus, we explored whether and how the components of place attachment mediate the relationship between local social identity and well-being.

## Materials and Methods

### Sample

#### Participants

We recruited 375 Italian participants (219 females, 156 males; mean age = 34.44; SD = 13.58, age range 18–87), living in cities (more than 5,000 inhabitants, 56%), small towns (<5,000 inhabitants, 26%), or rural areas (18%), by spreading an online survey. Participants took part in the survey on a voluntary basis.

### Procedure

An online questionnaire was implemented by using the Google Forms platform. Participants were recruited from different regions of Italy (mainly Lazio and Sicily) by university students for their Master's or Bachelor's thesis. Data were collected from March to November 2019.

The questionnaire took approximately 30 min to fill in. According to the ethical standards included in the Declaration of Helsinki (World Medical Association, 2001), participants were informed about all relevant aspects of the study (e.g., methods and institutional affiliations of the researchers) before they started to fill in the questionnaire. The research protocol was approved by the local ethics committee of the University of Rome “Sapienza” (October 29, 2018).

### Materials

The questionnaire included the following measures.

- *Satisfaction with Life*. Individual well-being of participants was assessed by using the *Satisfaction with Life Scale* (SWLS; Diener et al., 1985). The scale is comprised of five items that range from 1 (= It does not describe me at all) to 9 (= It describes me completely), (e.g., “Your life conditions are excellent”; α = 0.87, SWLS). Higher ratings indicate higher individual satisfaction with life.
- *Interdependent Happiness Scale* (IHS; Hitokoto and Uchida, 2015; Italian version, Mosca et al., 2021). The scale measures a relational aspect of well-being and consists of nine items that range from 1 (= It does not describe me at all) to 9 (=It describes me completely) (e.g., “You feel that you are positively evaluated by the others around you”; α = 0.82). Higher ratings indicate higher individual-interdependent happiness.
- *Place Attachment*. We have administered a slightly modified version of the PAHS (*Place Attachment to the Hometown Scale*) (Scopelliti and Tiberio, 2010). It included a 16-item self-report scale addressing physical, social, and functional aspects of attachment to the town or city of residence. Participants had to fill in the questionnaire referring to the city/village in which they lived and to indicate their opinion using a Likert scale ranging from 1 (= It does not describe me at all) to 9 (= It describes me completely). As described below, we carried out a factorial analysis to individuate the subdimensions of attachment to the city/village where people live measured on a sample not constituted only of university students, like in the originally published scale (Scopelliti and Tiberio, 2010). After having eliminated four items for statistical problems (see below), we extracted three subfactors^1^: (a) place identity (five items), measuring the degree of attachment with physical attributes of the attachment to city/village in which people live (e.g., The landscape of my city/village always makes me feel a strong emotion, α = 0.81); (b) social relations (three items), measuring a social aspect of the attachment to the place of residence (e.g., People I am attached to are mostly from my city/village, α = 0.68); (c) lack of resources (four items) (i.e., the reverse of place dependence), measuring a (dis)functional aspect of the attachment to the city/village in which people live (e.g., I often get bored there, α = 0.54, mean inter-item correlation =0.32^2^). Higher ratings indicate higher levels of place identity, quality of social relations in the place, and perception of lack of resources.
- *Local Social Identity Scale*. We administered a social identification *ad hoc* built scale (adapted from Paolini et al., 2020), composed of seven items to measure identification with the local community (e.g., Being part of the community of the people living in the city/village in which I live; is an important component for the image I have of myself; reflects well who I am; has to do with what I think about myself; bothers me; makes me feel good; α = 0.83). Participants had to report their answers on a Likert-type scale ranging from 1 (It doesn't describe me at all) to 9 (It describes me exactly). Higher ratings indicate stronger social identification with the local community.

### Statistical Analysis

Data analyses were performed with SPSS version 25, including the PROCESS model macro (Hayes and Preacher, 2014). PROCESS is a modeling tool that calculates the direct and indirect effects of mediation models, as well as the calculation of interactions and conditional indirect effects in moderation and moderated mediation models (see http://www.processmacro.org/index.html for more details). We conducted an exploratory factor analyses on the Place Attachment Scale because the original scale was validated on a student sample, while our sample was a community sample. Then, we calculated descriptive statistics and zero-order correlations between variables. Then, we conducted a series of mediation analyses with local social identity as an independent variable, satisfaction with life and interdependent happiness as dependent variables and place attachment subfactors, i.e., place identity, social relations, and lack of resources (i.e., reverse of place dependence) as mediators.

### Results

A principal components analysis with Promax rotation with Kaiser normalization was performed on the Place Attachment Scale. Scree plots were also used to confirm the expected number of factors and the factorial loading of each item in the expected component (i.e., subscale).

Four items saturated identically on two factors so they were removed for subsequent analysis (i.e., “I always know where to find what I look for there”; “I know how to feel relaxed there”; “The climate there makes me feel good”; “I feel proud to be part of my city/village”), and a new PCA with Promax rotation was conducted on 13 items. The Kaiser–Meyer–Olkin sampling adequacy measure attained fairly high values (= 0.86), demonstrating that communalities were high and the correlation matrix of the sample was appropriate for the analysis to proceed (Mundfrom et al., 2005). It yielded a three-factor solution explaining 57.1% of the variance (see Table 1). The factors were labeled according to the study of Scopelliti and Tiberio (2010), i.e., respectively, place identity, lack of resources, and social relations.

**Table 1:** Factor analysis for the place attachment scale.

	**Factor 1**	**Factor 2**	**Factor 3**
**Items**	**Place identity**	**Social relations**	**Lack of resources**
I like to know the history and traditions of my city/village	0.874		
My city/village is surrounded by many beautiful natural places	0.807		
I like to speak about my city/village to people which they don't know	0.731		
The landscape of my city/village always makes me feel a strong emotion	0.681		
Even if I would leave my city/village, it will be always a part of me	0.479		
People I am attached to are mostly from my city/village		0.892	
When I am away, I look forward coming back there to my friends		0.737	
When I am in my city/village I never feel alone		0.596	
I often get bored in my city-village			0.766
I always wanted to leave my city-village			0.759
I hardly found there people sharing my interests			0.641
My city/village offers lots of opportunities (R)			0.459
Eigenvalues	4.07	1.46	1.23
Explained Variance	33.92%	12.21%	10.24%

*R, reverse-coded*.

Descriptive statistics and zero-order correlations are reported in Table 2. Local social identity, place identity, and social relations were correlated positively with both satisfaction with life (Pearson's *r* ranging from 0.25 to 0.39, indicating a medium effect size) and interdependent happiness (Pearson's *r* ranging from 0.27 to 0.36, indicating equally a medium effect size). Lack of resources was correlated negatively with both SWL and IHS (*r* = −0.27, *p* < 0.01; *r* = −0.20, *p* < 0.01, respectively).

**Table 2:** Means, SD, skewness, kurtosis, and zero-order correlations (Pearson's *r*) between variables (*N* = 375).

	**Minimum**	**Maximum**	**Mean**	**SD**	**Sk**	**C**	**1**	**2**	**3**	**4**	**5**	**6**
1. SWLS	1.40	9.00	6.31	1.45	−0.72	0.53	1					
2. IHS	2.11	8.78	5.98	1.28	−0.32	−0.26	0.57^***^	1				
3. Local social identity	1.00	9.00	5.69	1.52	−0.30	0.30	0.39^***^	0.33^***^	1			
4. Place identity	1.40	9.00	7.34	1.43	−0.97	0.63	0.28^***^	0.27^***^	0.46^***^	1		
5. Social relations	1.00	9.00	6.14	1.87	−0.51	−0.19	0.32^***^	0.33^***^	0.53^***^	0.58^***^	1	
6. Lack of resources	1.00	9.00	4.88	1.59	0.09	−0.22	−0.27^***^	−0.20^***^	−0.45^***^	−0.30^***^	−0.30^***^	1

*N = 375;*

*** *p < 0.001; SWLS, Satisfaction with Life Scale; IHS, Interdependent Happiness Scale; Sk, skewness; C, kurtosis*.

### Mediation Analyses

In order to test our exploratory hypotheses, we tested different mediation models (PROCESS model number 4) with local social identity as the independent variable, satisfaction with life and interdependent happiness as the dependent variables, and place identity, social relations, and lack of resources (i.e., the subcomponents of Place Attachment) as mediators. Models 1, 2, and 3 tested the relationship between local social identity and satisfaction with life through place identity, social relations, and lack of resources, respectively. Models 4, 5, and 6 tested the relationship between local social identity and interdependent happiness through the same mediators of the previous analysis.

**Figure 1:**
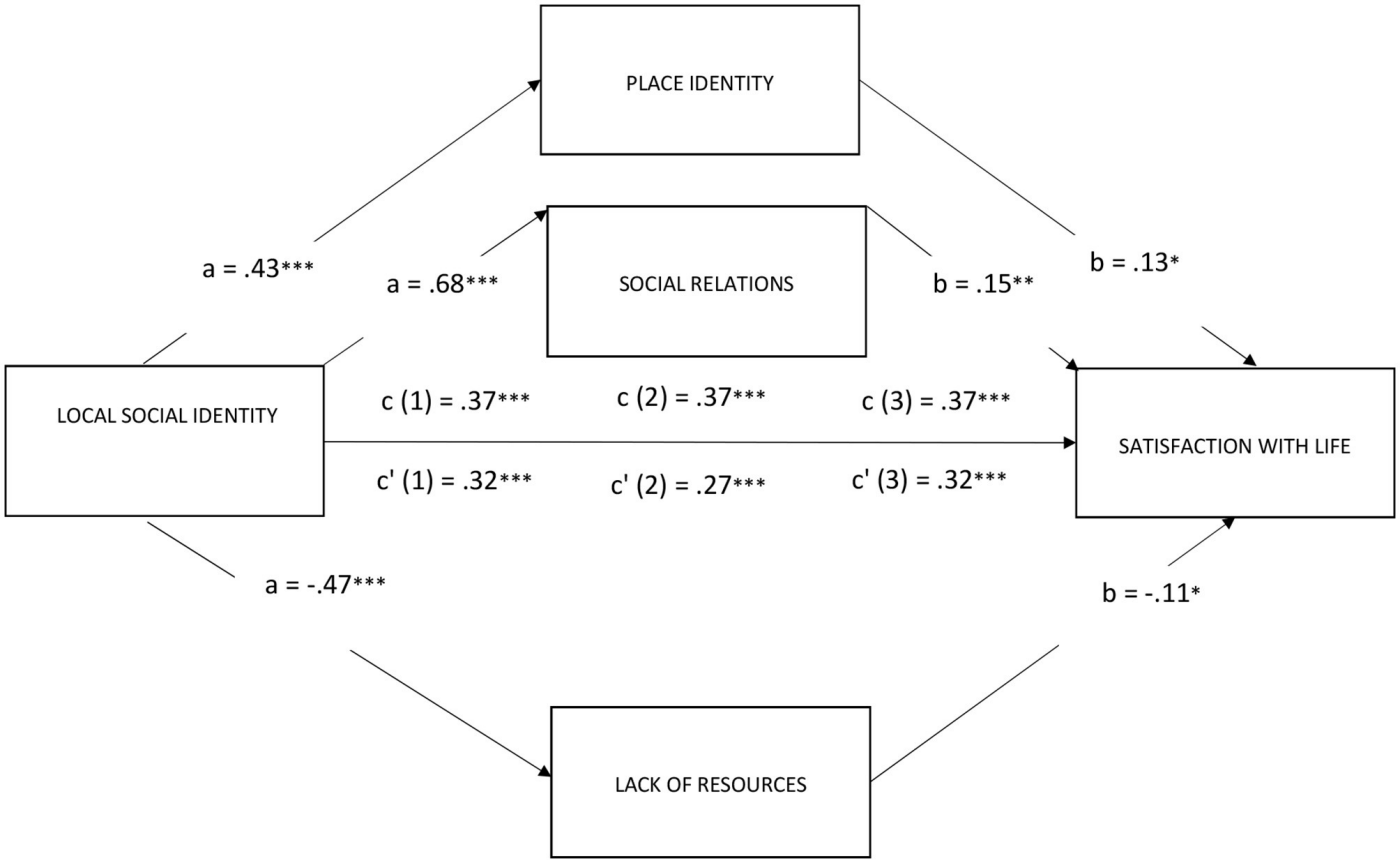
Place identity, social relations, and lack of resources mediate the effect of local social identity on satisfaction with life (***p < 0.001; **p < 0.01; *p < 0.05).

Models with satisfaction with life as dependent variable (see Figure 1).

Model 1: The overall equation was significant [*R*^2^ = 0.16; *F*_(2, 372)_ = 34.89, *p* < 0.001; see Figure 1]. The bootstrap analysis with 5,000 resampling showed the indirect effects of the local social identity of participants on their level of satisfaction with life via place identity (*b* = 0.0547; 95% CI: *LLCI* = 0.0073; *ULCI* = 0.1025) were significant. The direct effect considering the mediator was still significant (*b* = 0.3181; 95% CI: *LLCI* = 0.2106; *ULCI* = 0.4255). In other words, local social identity had a positive impact on satisfaction with life even after controlling for the indirect effects through place identity.

Model 2: The overall equation was significant [*R*^2^ = 0.17; *F*_(2, 372)_ = 40.13, *p* < 0.001; see Figure 1]. Indirect effects of the local social identity of participants on their level of satisfaction with life via social relations (*b* = 0.1033; 95% CI: *LLCI* = 0.0293; *ULCI* = 0.1768) were significant. The direct effect considering the mediator was still significant (*b* = 0.3728; 95% CI: *LLCI* = 0.2775; *ULCI* = 0.4680). In other words, local social identity had a positive impact on satisfaction with life even after controlling for the indirect effects through social relations.

Model 3: The overall equation was significant [*R*^2^ = 0.16; *F*_(2, 372)_ = 33.24, *p* < 0.001; see Figure 1]. Indirect effects of the local social identity of participants on their level of satisfaction with life via lack of resources (*b* = 0.0503; 95% CI: *LLCI* = −0.042; *ULCI* = −0.0994) were significant. The direct effect considering the mediator was still significant (*b* = 0.3225; 95% CI: *LLCI* = 0.2122; *ULCI* = 0.4328). In other words, local social identity had a positive impact on satisfaction with life even after controlling for the indirect effects through lack of resources.

**Figure 2:**
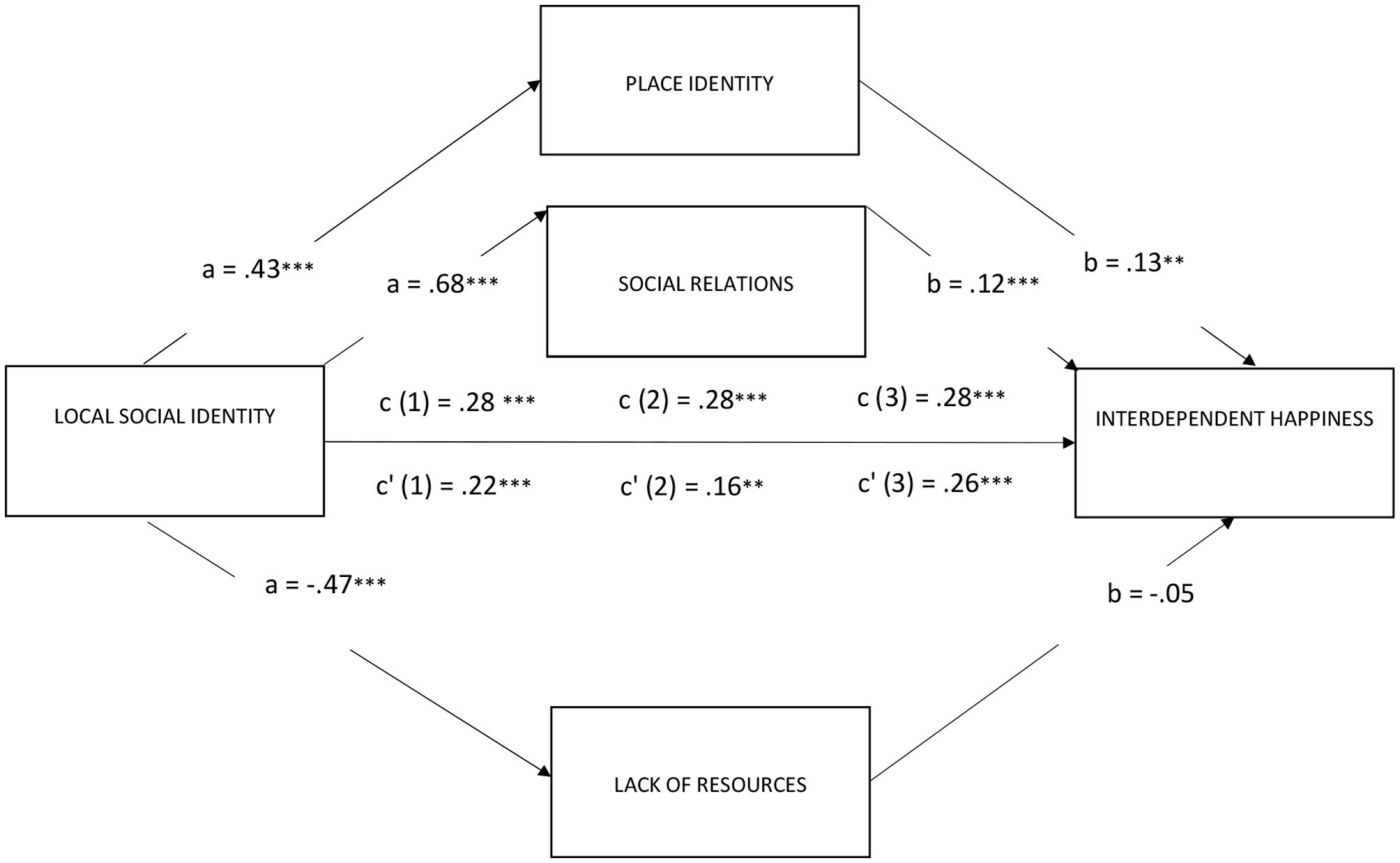
Place identity and social relations mediate the effect of local social identity on interdependent happiness (**p < 0.001; **p < 0.01; *p < 0.05).

Model 4: The overall equation was significant [*R*^2^ = 0.13; *F*_(2, 372)_ = 27.11, *p* < 0.001; see Figure 2]. The indirect effects of the local social identity of participants on their level of interdependent happiness via place identity (*b* = 0.0565; 95% CI: *LLCI* = 0.0204; *ULCI* = 0.0167) were significant. The direct effect considering the mediator was still significant (*b* = 0.2251.; 95% CI: *LLCI* = 0.1279; *ULCI* = 0.3223). In other words, local social identity had a positive impact on interdependent happiness even after controlling for the indirect effects through place identity.

Model 5: The overall equation was significant [*R*^2^ = 0.15; *F*_(2, 372)_ = 32.99, *p* < 0.001; see Figure 2]. The indirect effects of the local social identity of participants on their level of interdependent happiness via social relations (*b* = 0.1211; 95% CI: *LLCI* = 0.0611; *ULCI* = 0.1809) were significant. The direct effect considering the mediator was still significant (*b* = 0.1604; 95% CI: *LLCI* = 0.0561; *ULCI* = 0.2646); in other words, social identification with the local community had a positive impact on interdependent happiness even after controlling for the indirect effects through social relations. Model 6: The overall equation was significant [*R*^2^ = 0.11; *F*_(2, 372)_ = 21.32, *p* < 0.001; see Figure 2]. The indirect effects of social identification of participants with the local community on their level of interdependent happiness via lack of resources (*b* = 0.0242; 95% CI: *LLCI* = 0.0194; *ULCI* = 0.0675) were not significant.

## Discussion

The results of this study provide further evidence to the tripartite model of place attachment, consistent with other studies (Williams and Vaske, 2003; Scopelliti and Tiberio, 2010), and also to the consideration of place identity, place dependence, and social bonds as the key components of place attachment (e.g., Raymond et al., 2010; Ramkissoon et al., 2013; Chen et al., 2018). The study also corroborates the positive relationship between place attachment, local social identity, and relational and individual well-being. It is also shown how the dimensional distinction better explains the mediating role of place attachment factors in the relationship between local social identity and well-being.

Previous studies showed the links between social identification and well-being (e.g., Paolini et al., 2020), place attachment and well-being (e.g., Ratcliffe and Korpela, 2016, 2018), and community connectedness and activism (e.g., Rollero and De Piccoli, 2010). Moreover, previous studies demonstrated the mediating role of place attachment in the relationships between these variables (Buta et al., 2014). In this study, it is confirmed from the high relation between group identification and well-being (satisfaction with life and interdependent happiness), and from the importance of place attachment in connecting the strong identity bond of individuals with the local community which they belong to with their own well-being. This emerged taking into account not only the independent individual well-being, given by satisfaction with life (Diener et al., 1985), but also considering well-being as interdependent happiness, which is achieved with social relationships and harmony with others, in particular with the reference group of an individual (Hitokoto and Uchida, 2015).

Specifically, this study found that the relationship of local social identity with individual well-being (in terms of satisfaction with life and interdependent happiness) passes through the positive relationship with two dimensions of place attachment, i.e., place identity and social relations, while the perception of lack of resources (i.e., the reverse of place dependence) in the place where a person lives negatively mediates the relationship only between local social identity and satisfaction with life. This means that people with high local social identity develop a high identification with the place in terms of both the physical aspects of the place where they live (i.e., place identity) and the social relationships that they establish there; both these subdimensions of place attachment are positively related to individual well-being and interdependent happiness. Similarly, people with high local social identity have highly negative perceptions of the absence of resources in their place of living (in terms of functional attachment to the place), and this is negatively related to life satisfaction (but not to interdependent happiness). In other words, a high local social identity promotes a high place dependence, and this, in turn, is positively associated with life satisfaction but not with interdependent happiness.

### Limitations, Implications, and Future Research

This study has some limitations. In particular, it is a cross-sectional study (like most studies on place attachment and well-being), so it is not easy to clarify whether there is a causal direction of the relation between local social identity and place attachment. As pointed out in the literature review, these two psychosocial aspects are certainly highly interrelated. Future studies could manipulate the local identity degree of individuals to better understand the impact of place attachment on well-being. It is possible to think that these have a positive impact on the individual and relational well-being of people. That is, if one lives in a place with which she/he does not identify with or feels she/he does not belongs to, or to which she/he does not feel emotionally attached, then she/he does not experience satisfaction, well-being, or happiness either. On the other hand, social identification, place attachment, and well-being are psychological factors that mature over time, are bound to places, and are related to the social community. The merit of the present study lies in highlighting the role of place attachment in the relationship between local social identity and well-being, above all by investigating the different dimensions and facets of place attachment and their different impact on happiness and life satisfaction of people. Finally, most of our participants were female, not allowing us to test for the moderating role of the gender of participants. Future research would warrant a more in-depth investigation in this direction.

## Data Availability Statement

The raw data supporting the conclusions of this article will be made available by the authors upon request.

## Ethics Statement

This study was reviewed and approved by the Ethical Committee of the Department of Social and Development Psychology of Sapienza, University of Rome (October 29, 2018). Written informed consent for participation was not required for this study in accordance with the national legislation and the institutional requirements.

## Author Contributions

FM and OM contributed to data collection. OM and DP contributed to data analysis. FM and FF contributed to the interpretation and discussion of results. All the authors equally contributed to develop the project of the present research and to writing the paper.

## Conflict of Interest

The authors declare that the research was conducted in the absence of any commercial or financial relationships that could be construed as a potential conflict of interest.

## Publisher's Note

All claims expressed in this article are solely those of the authors and do not necessarily represent those of their affiliated organizations, or those of the publisher, the editors and the reviewers. Any product that may be evaluated in this article, or claim that may be made by its manufacturer, is not guaranteed or endorsed by the publisher.

## Footnotes

^1^For consistency reasons, we use in the Method and Results sections the factor labels used by the PAHS proposers (i.e., Scopelliti and Tiberio, 2010); nevertheless, we remind where appropriate along such sections that the subfactor “lack of resources” refers to “place dependence” in the theoretical approach we explicitly followed. It is to note that the factor label “lack of resources” has a negative sense; thus, high scores mean low “place dependence,” and low scores mean high “place dependence.”

^2^Due to the fact that Cronbach's alpha values are sensitive to the number of items of the scale, when such a number is low, it is common to find quite low Cronbach's alpha values as in this case. For this reason, we have reported the mean inter-item correlation of the items, and those results are included in the optimal range for the inter-item correlation (i.e., between 0.20 and 0.40) recommended by Briggs and Cheek (1986).
